# Non-COVID-19 respiratory tract infection mortality among children under 5 years old: a time series, Brazil, 2002-2022

**DOI:** 10.1590/S2237-96222026v35e20250575.en

**Published:** 2026-04-10

**Authors:** Márcia de Cantuária Tauil, André Peres Barbosa de Castro, Silvânia Suely Caribé de Araújo Andrade, Felipe Teixeira de Mello Freitas, Noely Fabiana Oliveira de Moura

**Affiliations:** 1 Centro Universitário Euroamericano, Curso de Medicina, Brasília, DF, Brazil; 2 Ministério da Saúde, Secretaria de Gestão do Trabalho e da Educação na Saúde, Brasília, DF, Brazil; 3 Defensoria Pública da União, Brasília, DF, Brazil; 4 Escola Superior de Ciências da Saúde, Curso de Medicina, Brasília, DF, Brazil; 5 Fundação Oswaldo Cruz, Núcleo de Epidemiologia e Vigilância em Saúde, Brasília, DF, Brazil

**Keywords:** Respiratory Tract Infections, Child, Mortality Information System, Time Series, Brazil, Infecciones del Sistema Respiratorio, Niño, Sistema de Información sobre Mortalidad, Series de Tiempo, Brasil

## Abstract

**Objectives:**

To describe and analyze the temporal trend of non-COVID-19 respiratory tract infection deaths among children under 5 years of age in Brazil from 2002 to 2022.

**Methods:**

This is a descriptive study of respiratory tract infection deaths and time series analysis of mortality rates using data from the Mortality Information System. Trend analysis used the Trigonometric seasonality, Box-Cox transformation, ARMA errors, Trend and Seasonal components model for interrupted time series, examining monthly mortality rates. Annual percentage change (APC) for the period was estimated in order to present the time series trend and its 95% confidence interval (95%CI).

**Results:**

A total of 52,015 respiratory tract infection deaths were recorded among children under 5 years old during the period, with a higher incidence among males (54.2%), Black children (46.0%), and those under 1 year of age (66.9%). The respiratory tract infection mortality rate among children under 5 years old in Brazil in 2002 was 1.22 deaths/1,000 live births, and in 2022 it was 0.87 deaths/1,000 live births. The proportional mortality rate in children aged 28 days to 5 years varied around 13.0%. There was a decreasing mortality rate trend from 2002 to 2019 (APC -0.37%; 95%CI -0.43; -0.31). In the post-pandemic period, there was no significant variation in the mortality rate analyzed (APC -1.41%; 95%CI -8.74; 6.52).

**Conclusion:**

Despite the reduction in respiratory infection mortality rates along the time series, there was no reduction in proportional mortality. New strategies are needed to prevent and reduce respiratory tract infection deaths in the Brazilian health system.

## Resumen

**Objetivos:**

Describir y analizar la tendencia temporal de las muertes por infecciones respiratorias no relacionadas con COVID-19 en niños menores de 5 años en Brasil, entre 2002 y 2022.

**Métodos:**

Estudio descriptivo de las muertes por infecciones respiratorias y análisis de series temporales de las tasas de mortalidad utilizando datos del Sistema de Información de Mortalidad. El análisis de tendencias utilizó el modelo Trigonometric seasonality, Box-Cox transformation, ARMA errors, Trend and Seasonal components para series temporales interrumpidas, examinando las tasas de mortalidad mensuales. Se estimó la variación porcentual anual (VPA) para el período para presentar la tendencia de la serie y su intervalo de confianza del 95% (IC95%).

**Resultados:**

Se registraron 52.015 muertes por infecciones respiratorias en niños menores de 5 años durante el período, con mayor incidencia entre los varones (54,2%), los niños negros (46,0%) y los menores de 1 año (66,9%). La tasa de mortalidad por infecciones respiratorias en niños menores de 5 años en Brasil en 2002 fue de 1,22 muertes/1.000 nacidos vivos, y en 2022 fue de 0,87 muertes/1.000 nacidos vivos. La tasa de mortalidad proporcional en niños de 28 días a 5 años varió alrededor del 13,0%. Se observó una tendencia decreciente en las tasas de mortalidad entre 2002 y 2019 (VPA -0,37%; IC95% -0,43; -0,31). En el período pospandémico, no se observó variación significativa en la tasa de mortalidad analizada (VPA -1,41%; IC95% -8,74; 6,52).

**Conclusión:**

A pesar de la reducción en las tasas de mortalidad por estas enfermedades a lo largo de la serie histórica, no se ha observado una reducción en la mortalidad proporcional. Se necesitan nuevas estrategias para prevenir y reducir las muertes por infecciones respiratorias en el sistema de salud brasileño.

**Ethical aspects**

This research used public domain anonymized databases.

## Introduction 

From 2010 to 2020, there was a 23% reduction in mortality among children under 5 years of age in Brazil (1). This occurred mainly in the post-neonatal component (28-364 days of life) and in early childhood (1-4 years), including a reduction in respiratory disease mortality, influenced by high *Haemophilus influenzae* vaccination coverage and the introduction of the *Streptococcus pneumoniae* vaccine in 2009 (1,2). However, mortality among children under 5 years old in Brazil was considered high when compared to high-income countries. Respiratory diseases were the fifth leading cause of death in children under 5 years of age until 2019, being the leading cause, excluding the neonatal period (3).

Between 2008 and 2012, in Ecuador and the United States – countries that, like Brazil, implemented vaccination against *Streptococcus pneumoniae* – viruses were identified as the main cause of respiratory infection, with 70% of cases in children under 5 years old. Respiratory syncytial virus was the main agent, especially among children under 1 year old, commonly being the main etiological agent of bronchiolitis (4,5).

In 2014 in Latin America, a 1% case fatality ratio was identified among respiratory infections caused by respiratory syncytial virus (6). It is estimated that respiratory syncytial virus causes 33 million lower respiratory tract infections annually in children under 5 years old globally, resulting in 3.6 million hospitalizations and 26,300 deaths (7). There are no estimates of the number of deaths caused by respiratory syncytial virus in Brazil.

In October 2023, the Brazilian National Health Surveillance Agency approved a new single annual monoclonal antibody dose against respiratory syncytial virus and, in April 2024, it approved a new respiratory syncytial virus vaccine for pregnant women, with the aim of preventing infections by this virus in early childhood. In February 2025, both were incorporated into the Brazilian Unified Health System (8,9).

This study aimed to describe and analyze the temporal trend of non-COVID-19 respiratory infection deaths in children under 5 years of age in Brazil from 2002 to 2022. It is expected to serve as a baseline for future studies, following the widespread adoption of new respiratory syncytial virus prevention technologies.

## Methods 

### Design 

This is a time series study of non-COVID-19 respiratory infection mortality rates among children under 5 years of age in Brazil, from 2002 to 2022, in addition to a description of these deaths.

### Setting 

Brazil is a country divided into five regions (North, Northeast, Midwest, Southeast and South) with 27 Federative Units. According to the Brazilian Institute of Geography and Statistics, the country had 214,828,540 inhabitants in 2022. In the same year, 2,561,922 children were born alive, and there were 14,675,523 children under 5 years old, which corresponded to 1.19% and 6.83% of the total population, respectively (10,11).

### Participants 

We took into consideration deaths of children under 5 years old due to respiratory infection reported on the Mortality Information System with the underlying cause recorded under the following 10th revision of the International Classification of Diseases codes: B97 (viral agents as the cause of diseases classified in other chapters), J00-J06 (acute upper respiratory infections), J09-J18 (influenza and pneumonia), J20-J22 (other acute lower respiratory infections), or J40 (bronchitis, not specified as acute or chronic). Deaths with an underlying cause related to COVID-19 were not included.

### Variables 

Non-COVID-19 respiratory infection deaths were described according to date of death, place of birth, age at death in years (0, 1, 2, 3, 4), sex (female, male, unknown), birth weight (<2,500 g; ≥2,500 g), race/skin color (Asian, White, Indigenous, Black [Black and Brazilian mixed race], unknown), place of death (hospital, other health service, home, other), maternal age group (<15, 15-19, 20-24, 25-29, 30-34, ≥35 years), maternal education (none, 1-3, 4-7, 8-11, 12+ years, unknown), number of children alive (≤1, 2-3, >3, unknown), type of delivery (cesarean, vaginal, unknown), type of pregnancy (single, twin, triplet, and more, ignored) and weeks of gestation (≤36 weeks, >36 weeks, unknown).

We calculated the epidemiological indicators described below.

- Non-COVID-19 respiratory infection mortality rate among children under 5 years old in Brazil/region: non-COVID-19 respiratory infection deaths among children under 5 years old in Brazil/region divided by the number of live births in Brazil/region multiplied by 1,000.
- Non-COVID-19 respiratory infection proportional mortality among children under 5 years old in Brazil/region: non-COVID-19 respiratory infection deaths among children under 5 years old in Brazil/region divided by the total number of deaths in children under 5 years old in Brazil/region multiplied by 100.

### Data sources

The Mortality Information System data were obtained from the Oswaldo Cruz Foundation Health Data Science Platform (12). This Platform used anonymized data made available by the Brazilian Unified Health System Department of Information Technology, performing processing and enrichment via its own methodology (13).

We also used the live birth numbers held on the Live Birth Information System, available from the Brazilian Unified Health System Department of Information Technology website, via Tabnet, for Brazil and its Federative Units according to the years covered by the study (11). The database and analysis codes used in the research are available at: https://drive.google.com/drive/folders/17aHqCAwu3j2qjjb97wUd3jbeIV4Vq_XF?usp=sharing (14).

### Statistical methods

The descriptive statistics for deaths in this study were estimated using absolute and relative frequencies of the selected variables. The epidemiological indicators mentioned were calculated annually for both Brazil as a whole and for its geographic regions. With regard to the time series analyses, non-COVID-19 respiratory infection mortality rates among children under 5 years old were calculated monthly for Brazil as a whole and we applied the Trigonometric seasonality, Box-Cox transformation, ARMA errors, Trend and Seasonal components (TBATS) model, part of the Forecast package of the R computing environment (version 4.3.1, R Foundation for Statistical Computing, Vienna, Austria. Available at: https://www.R-project.org/) (15). 

The TBATS model was considered adequate after assessing residual autocorrelation using an autocorrelation function plot and the Ljung-Box test (16). In addition, the Seastests package was used to identify trends and seasonality using the Friedman test (17). The interrupted time series analysis method was used due to the occurrence of the COVID-19 pandemic in 2020 and 2021 (17).

We analyzed monthly non-COVID-19 respiratory infection mortality rates in children under 5 years old (dependent variable) for the period 2002-2022 (independent variable), with a 12-month seasonality pattern. The years in the series prior to the COVID-19 pandemic were 2002 to 2019, while the pandemic period was between 2020 and 2021. The year 2022 was considered to be post-COVID-19 pandemic (18).

In order to describe linear trends, a p-value<0.05 indicated statistical significance. Annual percentage change (APC) over the period was estimated to present the trend of the series by calculating the natural logarithm of the monthly non-COVID-19 respiratory infection mortality rates among in children under 5 years old in order to linearize the trend. A linear regression model was applied, and APC was calculated using the following equation (18): APC=(exp(β)−1)*100, where β is the regression coefficient. 

The APC 95% confidence intervals da APC were also estimated using the formula IC_APC_=(exp(β±t_α_/_2_,_df_×SE(β))−1*100, where t_α_/_2_,_df_ is the critical value of the T test with a 5% significance level and df are the model’s degrees of freedom (number of observations less number of parameters). 

The trend was considered to be rising when APC values were positive, falling when APV values were negative, and stable when the results were not statistically significant.

## Results 

Between 2002 and 2022, 52,015 deaths among children under 5 years old Brazil, with non-COVID-19 respiratory infection as the underlying cause, were recorded on the Mortality Information System. The highest frequencies of death were observed in: male children (54.2%; n=28,174), Black children (46.0%; n=23,936), children under 1 year old (66.9%; n=34,792), and hospital as the place of death (85.0%; n=44,197) (Table 1). There was a higher percentage of deaths of White children in the Southeast (54.4%; n=9,640) and South (81.9%; n=3,421). Similar to the birth weight variable, more than 50.0% of the information was missing for the maternal variables age group, education level, type of pregnancy, gestational age, type of delivery and number of children alive.

**Table 1:** Characteristics of non-COVID-19 respiratory tract infection deaths among children under 5 years old. Brazil and its regions, 2002-2022 (n=52,015)

Variables	Brazil n (%)	North n (%)	Northeast n (%)	Midwest n (%)	Southeast n (%)	South n (%)
Sex						
Female	23,813 (45.8)	4,366 (45.7)	7,609 (45.7)	1,787 (45.6)	8,164 (46.1)	1,887 (45.2)
Male	28,174 (54.2)	5,173 (54.2)	9,031 (54.2)	2,131 (54.4)	9,551 (53.9)	2,288 (54.8)
Unknown	28 (0.1)	7 (0.1)	17 (0.1)	2 (0.0)	2 (0.0)	0 (0.0)
**Age** (years)						
0	34,792 (66.9)	6,392 (67.0)	11,204 (67.3)	2,550 (65.0)	11,814 (66.7)	2,832 (67.9)
1	9,156 (17.6)	1,710 (17.9)	2,934 (17.6)	716 (18.3)	3,077 (17.4)	719 (17.2)
2	3,953 (7.6)	736 (7.7)	1,197 (7.2)	344 (8.8)	1,374 (7.7)	302 (7.2)
3	2,418 (4.6)	438 (4.6)	763 (4.6)	190 (4.8)	847 (4.8)	180 (4.3)
4	1,696 (3.3)	270 (2.8)	559 (3.3)	120 (3.1)	605 (3.4)	142 (3.4)
**Race/skin color**						
Asian	111 (0.2)	22 (0.2)	36 (0.2)	7 (0.2)	41 (0.2)	5 (0.1)
Indigenous	2,482 (4.8)	1,393 (14.6)	251 (1.5)	659 (16.8)	74 (0.4)	105 (2.5)
White	20,452 (39.3)	1,986 (20.8)	3,972 (23.8)	1,433 (36.5)	9,640 (54.4)	3,421 (81.9)
Black	23,936 (46.0)	5,737 (60.1)	9,521 (57.2)	1,571 (40.1)	6,638 (37.5)	469 (11.3)
Unknown	5,034 (9.7)	1,393 (4.3)	2,877 (17.3)	250 (6.4)	74 (7.5)	175 (4.2)
**Place of occurrence of death**						
Hospital	44,197 (85.0)	7,914 (82.9)	14,010 (84.1)	3,274 (83.5)	15,428 (87.1)	3,571 (85.5)
Other health service	1,931 (3.7)	161 (1.7)	351 (2.1)	154 (3.9)	1,149 (6.5)	116 (2.8)
Household	4,079 (7.8)	921 (9.6)	1,598 (9.6)	321 (8.2)	891 (5.0)	348 (8.3)
Other places	1,641 (3.2)	487 (5.1)	632 (3.8)	162 (4.1)	231 (1.3)	129 (3.1)
Unknown	167 (0.3)	63 (0.7)	66 (0.4)	9 (0.2)	18 (0.1)	11 (0.3)

**Figure 1:**
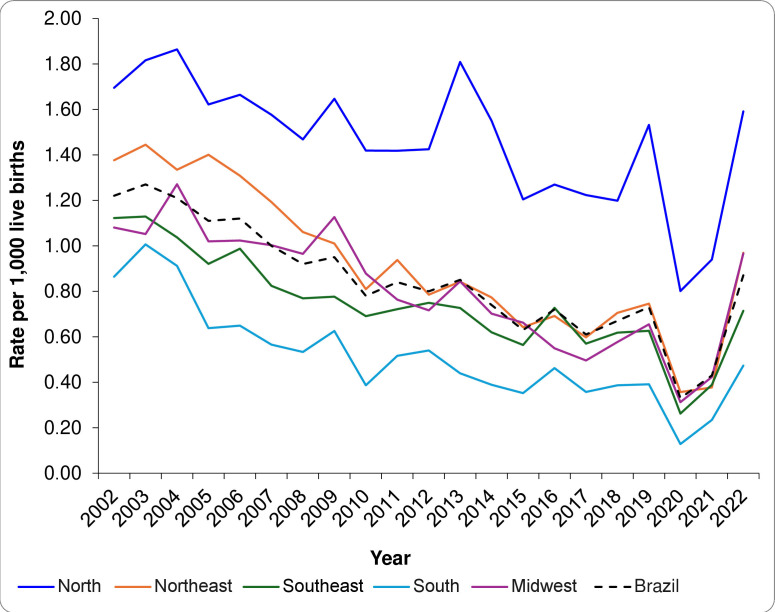
Non-COVID-19 respiratory tract infection mortality rate among children under 5 years old. Brazil and its regions, 2002-2022

The non-COVID-19 respiratory infection mortality rate among children under 5 years old in Brazil in 2002 was 1.22 deaths per 1,000 live births, while in 2022 it was 0.87 deaths per 1,000 live births. The lowest mortality rate was recorded in 2020 (0.33 per 1,000 live births) (Table 2). The North had the highest mortality rate per 1,000 live births throughout the period, with the highest recorded in 2004 (1.86 deaths per 1,000 live births). The South had the lowest rate with 0.13 deaths per 1,000 live births in 2020 (Figure 1).

**Table 2:** Non-COVID-19 mortality rate and proportional mortality among children under 5 years old. Brazil, 2002-2022 (n=52,015)

Year	Mortality rate among children under 5 years old per 1,000 live births	Proportional mortality (%) among children under 5	Proportional mortality (%) among children under 5, excluding neonatal deaths (n=50,159)
2002	1.22	5.68	12.86
2003	1.27	6.01	13.40
2004	1.21	6.09	14.02
2005	1.11	5.91	13.33
2006	1.12	6.01	14.77
2007	1.00	5.67	13.48
2008	0.92	5.45	13.34
2009	0.95	5.71	13.88
2010	0.78	4.98	12.58
2011	0.84	5.50	13.55
2012	0.80	5.37	13.23
2013	0.85	5.71	13.31
2014	0.74	5.18	13.03
2015	0.63	4.65	12.22
2016	0.72	5.11	12.64
2017	0.61	4.46	11.51
2018	0.67	4.96	12.43
2019	0.73	5.29	13.78
2020	0.33	2.66	6.93
2021	0.43	3.31	8.71
2022	0.87	6.11	14.92

**Figure 2:**
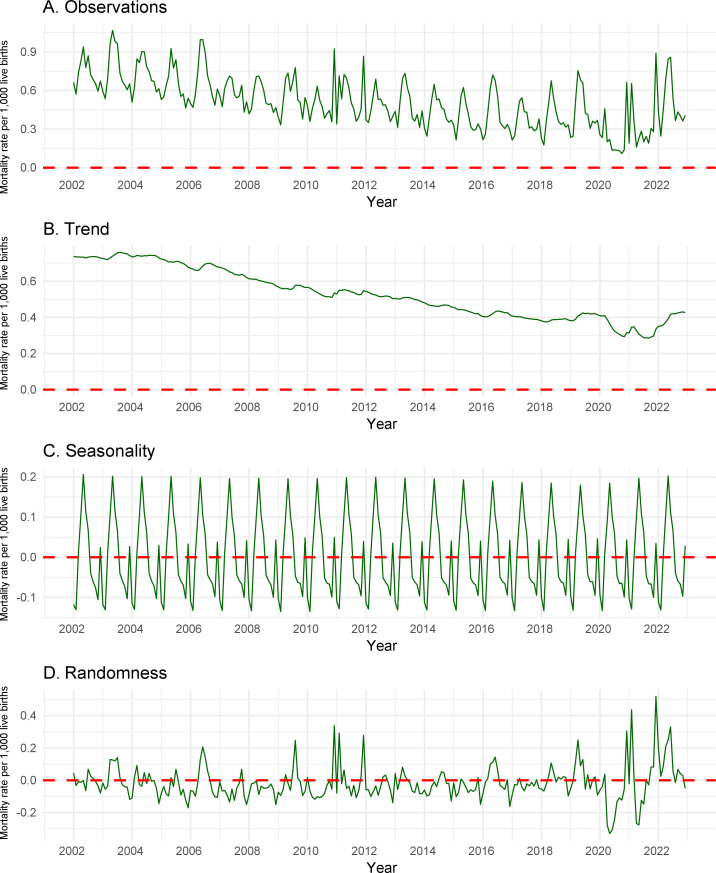
Time series of the monthly mortality rate (per 1,000 live births) due to non-COVID-19 respiratory tract infection in children under 5 years old: (A) observations; (B) trend; (C) seasonality; (D) randomness. Brazil, 2002-2022

Between 2002 and 2022, non-COVID-19 respiratory infection mortality among children in Brazil showed annual seasonality, with an increase in the mortality rate in mid-March, peaking in May and decreasing in July. An increase in the rate was also observed in January followed by a decrease. The seasonal pattern was maintained during the pre-COVID-19 pandemic period and, from 2020 onwards, annual seasonality was observed, but with smaller peaks in mortality rates (Figure 2).

Figure 2 presents the distribution of the monthly non-COVID-19 respiratory infection mortality rate (Figure 2A), the mortality rate trend (Figure 2B), and the seasonal pattern of mortality rate occurrence (Figure 2C). Randomness (or white noise) specified the behavior of the non-COVID-19 respiratory infection mortality rate among children under 5 years old not explained by trend or seasonality (Figure 2D).

Between 2002 and 2022, an average annual reduction of 0.01 was observed in the non-COVID-19 respiratory infection mortality rate among children under 5 years old, and this trend was considered to be statistically significant (p-value 0.034). Considering the interrupted time series analysis (interruption during the pandemic in 2020 and 2021), there were two study periods: 2002-2019 and the year 2022.

A downward trend in the mortality rate was observed in the period 2002-2019 (APC -0.37%; 95%CI -0.43; -0.31). The pandemic years 2020 and 2021 presented the lowest mortality rates in the series, affecting the behavior of the mortality rate throughout the years analyzed. In 2022 (post-pandemic), there was no significant variation in the non-COVID-19 respiratory infection mortality rate among children under 5 years old (APC -1.41%; 95%CI -8.74; 6.52) (Table 3).

**Table 3:** Annual percentage change (APC) and 95% confidence interval (95%CI) of the non-COVID-19 respiratory tract infection mortality rate among children under 5 years old. Brazil, 2002-2022 (n=52,015)

Period (years)	APC	95CI%
2002-2019	-0.37%	-0.43; -0.31
2022	-1.41%	-8.74; 6.52

Proportional mortality during the study period ranged from 2.7% in 2020 to 6.1% in 2022 for the country as a whole (Table 2). The regions with the lowest and highest proportional non-COVID-19 respiratory infection mortality during the study period were the South in 2020 (1.3%) and the North in 2023 (9.6%) (data not shown in tables or figures). When excluding the neonatal period, proportional mortality in children aged 28 days to 5 years varied around 13.0% per year, with the lowest value being 11.5% in 2017 and the highest 14.9% in 2022. In the pandemic years, this rate was 6.9% in 2020 and 8.7% in 2021 (Table 2).

## Discussion 

In Brazil, between 2002 and 2022, there was a higher concentration of respiratory infection deaths among children under 1 year old and among Black children. Non-COVID-19 respiratory infection mortality rates among children under 5 years old were higher in the North of the country and lower in the South in all years studied. The proportional mortality rate among children aged 28 days to under 5 years was above 13%. Throughout the period, annual seasonality was identified in Brazil as a whole, with a downward trend in the respiratory infection mortality rate.

This study showed that respiratory infection mortality among children under 5 years of age in Brazil was within the target of less than 3 deaths per 1,000 live births by 2025 established by the World Health Organization and the United Nations Children’s Fund in their Global Action Plan for Pneumonia and Diarrhea, which aims to end preventable deaths from pneumonia (19). A significant decrease in respiratory infection mortality in children under 5 years old was observed in Brazil from 2002 to 2019, this period being prior to the COVID-19 pandemic. The decrease in non-COVID-19 respiratory infection deaths was even more pronounced during the COVID-19 pandemic in 2020 and 2021, likely reflecting the effect of non-pharmaceutical COVID-19 prevention measures, such as school closures, social isolation and widespread use of masks in public settings. In 2022, there was an increase in the mortality rate among children under 5 years old to levels close to those observed in 2019, once COVID-19 prevention measures had been lifted.

Global burden of disease study estimates showed a significant decrease, of 67% and 77%, in deaths from respiratory infections among children under 5 years of age from 1990 to 2019, worldwide and in Latin America, respectively (20). Data from 2021 estimated the respiratory infection mortality rate among children under 5 years of age per 100,000 inhabitants at 76 globally and 25 for Latin America (20). In Brazil, this reduction was 96% from 1980 to 2021, going from 302 to 11 deaths among children under 5 years of age due to pneumonia and other respiratory infections per 100,000 inhabitants, respectively (20). Much of this reduction globally and in Brazil had been attributed to measures such as promoting exclusive breastfeeding until 6 months of age, increasing *Haemophilus influenzae* and *Streptococcus pneumoniae* vaccination coverage, increasing access to health services, and appropriate clinical management of respiratory infection cases (21).

Despite all this historical progress, respiratory infection mortality is related to levels of poverty and inequality (20). Respiratory disease mortality is the second leading cause of death in children under 5 years old globally and was the leading cause of death in childhood excluding neonatal causes in Brazil in 2019 (1,22).

Regarding proportional respiratory infection mortality, it accounts for more than 10% of deaths excluding the neonatal period. In Brazil, there was no reduction in proportional mortality during the study period, except during the COVID-19 pandemic period, which shows a proportionally smaller reduction in deaths due to respiratory infection when compared to other causes.

The majority of deaths occurred among Black children. Infant mortality due to preventable causes affects Black children more, and this factor is also associated with low maternal education (23). In Brazil, among children born between January 1, 2012, and December 31, 2018, Black children had a 39% higher risk of dying before the age of 5 compared to White children, in addition to a 78% increased risk of death due to pneumonia (24). Factors such as limited access to health services, poor sanitation conditions and higher malnutrition incidence were identified as factors contributing to this disparity.

The strongest estimates of association between maternal race/skin color and infant mortality were found for influenza and pneumonia. The greatest ethnic-racial disparities were observed in children aged 1 to 4 years regarding diarrhea, malnutrition, influenza and pneumonia (24). Survival of post-neonates and children aged 1 to 4 years was most adversely affected by family living conditions, income, education, basic sanitation, access to clean water and access to health services (24-26).

The North stood out for having the highest values ​​throughout the entire time series, unlike the South. The South had the highest percentage of the population that reported having had at least one medical and dental consultation during the year when compared to other regions of the country (27). The North faced challenges such as lower primary care coverage, greater distance from specialized health services, and a shortage of pediatric and intensive care unit beds (28). These factors limited adequate management of severe respiratory infection cases. On the other hand, the South benefited from higher human development indicators, a greater number of well-equipped hospitals, and better distribution of health professionals, which contributed to the reduction in mortality rates (28,29).

Respiratory disease mortality seasonality was observed in all the years of the time series, beginning in March, peaking in May and decreasing in July. This seasonal pattern of respiratory disease mortality coincided with respiratory syncytial virus seasonality, this being the main respiratory infection agent in children under 5 years old, especially in children under 1 year old (30).

Interpretation of the results of this study needs take into account limitations related to the use of secondary data, which may present underreporting and typing errors. There was a high percentage of variables with missing categories, such as the child’s birth weight and those related to mothers (age range, education level, gestational age, type of delivery and number of children alive). These are important variables, but it was not possible to interpret them, and they may be underestimated or overestimated. Therefore, it is important to raise awareness among health professionals responsible for this information so that all death certificate variables are completed.

It is possible that some respiratory disease deaths were not correctly classified on the death certificates and on the Mortality Information System; this may lead to underestimation of the number of deaths. However, considering that the data series is long and that improvements have been made to the Mortality Information System in recent years, it is possible that, in more recent years, deaths are now better classified than in previous years.

In conclusion, this study identified respiratory infection mortality rates in Brazil using secondary data from the national mortality surveillance system. Occurrence of deaths appears to follow seasonality that coincides with that of respiratory syncytial virus, the main agent of severe respiratory infection in children under 5 years old. Despite the reduction in mortality rates from these diseases throughout the time series, there was no reduction in proportional mortality, which highlights that new strategies are needed to prevent and reduce respiratory infection deaths in the Brazilian health system.

These results gain relevance since they serve as a basis for evaluating the impact of new technologies for preventing respiratory syncytial virus infections incorporated into the Brazilian Unified Health System: a vaccine for pregnant women and a new monoclonal antibody to protect against respiratory syncytial virus. A higher number of deaths were observed in Black children and in the North of the country, which may reflect inequalities in the prevention and treatment of these infections. These data are only descriptive; therefore, future analytical studies are needed to assess the risk related to these variables.
