# Supplementary material for: Non-COVID-19 respiratory tract infection mortality among children under 5 years old: a time series, Brazil, 2002-2022
Source: Epidemiol Serv Saude. 2026 Apr 10;35:e20250575. doi: 10.1590/S2237-96222026v35e20250575.en (PMC13073076; doi:10.1590/S2237-96222026v35e20250575.en)
Supplement: Translation

# Translation: Mortalidade por infecções respiratórias não covid-19 em menores de 5 anos de idade: série temporal, Brasil, 2002-2022

**DOI:** 10.1590/S2237-96222026v35e20250575.pt

**Authors:** Márcia de Cantuária Tauil, André Peres Barbosa de Castro, Silvânia Suely Caribé de Araújo Andrade, Felipe Teixeira de Mello Freitas, Noely Fabiana Oliveira de Moura

**Affiliations:** 1 Centro Universitário Euroamericano, Curso de Medicina, Brasília, DF, Brasil; 2 Ministério da Saúde, Secretaria de Gestão do Trabalho e da Educação na Saúde, Brasília, DF, Brasil; 3 Defensoria Pública da União, Brasília, DF, Brasil; 4 Escola Superior de Ciências da Saúde, Curso de Medicina, Brasília, DF, Brasil; 5 Fundação Oswaldo Cruz, Núcleo de Epidemiologia e Vigilância em Saúde, Brasília, DF, Brasil

**Keywords:** Infecções Respiratórias, Criança, Sistema de Informações sobre Mortalidade, Série Temporal, Brasil

## Abstract

**Objetivos:**

Descrever e analisar a tendência temporal dos óbitos por infecções respiratórias não covid-19 em crianças menores de 5 anos no Brasil de 2002 a 2022.

**Métodos:**

Estudo descritivo dos óbitos por infecção respiratória e análise de série temporal das taxas de mortalidade com dados do Sistema de Informações sobre Mortalidade. Análise de tendência utilizou o modelo Trigonometric Box-Cox Transformation ARMA errors Trend and Seasonal Components para séries temporais interrompidas examinando as taxas mensais de óbitos. Estimou-se a variação percentual anual (VPA) do período para apresentar a tendência da série e seu intervalo de confiança de 95% (IC95%).

**Resultados:**

Registraram-se 52.015 óbitos por infecções respiratórias em crianças menores de 5 anos no período, sendo maior no sexo masculino (54,2%), negros (46,0%) e menores de 1 ano de idade (66,9%). A taxa de mortalidade por infecções respiratórias em menores de 5 anos no Brasil em 2002 foi 1,22 óbito/1 mil nascidos vivos e em 2022 foi 0,87 óbito/1 mil nascidos vivos. A mortalidade proporcional em crianças de 28 dias a 5 anos variou em torno de 13,0%. Houve tendência de redução da taxa de mortalidade de 2002 a 2019 (VPA -0,37%; IC95% -0,43; -0,31). No pós-pandemia, não houve variação significativa na taxa de mortalidade analisada (VPA -1,41%; IC95% -8,74; 6,52).

**Conclusão:**

Apesar da redução das taxas de mortalidade por essas doenças ao longo da série histórica, não houve redução da mortalidade proporcional. Novas estratégias são necessárias para prevenir e reduzir os óbitos por infecções respiratórias no sistema de saúde brasileiro.

**Aspectos éticos**

Esta pesquisa utilizou bancos de dados de domínio público e anonimizados.:

## Introdução 

De 2010 a 2020, houve redução de 23% da mortalidade em menores de 5 anos no Brasil (1). Isso se deu principalmente no componente pós-neonatal (28-364 dias de vida) e na infância (1-4 anos), incluindo redução na mortalidade por doenças respiratórias, influenciada pela alta cobertura de vacinação contra *Haemophilus influenzae* e pela introdução da vacina contra *Streptococcus pneumoniae* em 2009 (1,2). No entanto, a mortalidade em menores de 5 anos no Brasil foi considerada alta quando comparada com países de alta renda. As doenças respiratórias respondiam como a quinta causa de óbito em menores de 5 anos até 2019, sendo a primeira causa, excluído o período neonatal (3).

Entre 2008 e 2012, no Equador e nos Estados Unidos – países que, assim como o Brasil, implementaram a vacinação contra *Streptococcus pneumoniae* –, identificou-se vírus como a principal causa de infecção respiratória, 70% dos casos em menores de 5 anos. O vírus sincicial respiratório foi o principal agente, em especial entre crianças menores de 1 ano, sendo comumente o principal agente etiológico de bronquiolite (4,5). 

Em 2014 na América Latina, identificou-se a taxa de letalidade de 1% entre os casos de infecção respiratória causada por vírus sincicial respiratório (6). Estima-se que o vírus sincicial respiratório cause anualmente 

33 milhões de infecções do trato respiratório inferior em crianças com menos de 5 anos no mundo, o que resulta em 3,6 milhões de hospitalizações e 26,3 mil óbitos (7). Não há estimativas do número de óbitos causados por vírus sincicial respiratório no Brasil.

Em outubro de 2023, a Agência Nacional de Vigilância Sanitária do Brasil aprovou um novo anticorpo monoclonal de dose única anual contra o vírus sincicial respiratório e, em abril de 2024, uma nova vacina para gestantes contra vírus sincicial respiratório, com o intuito de prevenir as infecções por esse vírus na primeira infância. Em fevereiro de 2025, ambos foram incorporados no Sistema Único de Saúde (8,9). 

Este estudo teve como objetivos descrever e analisar a tendência temporal dos óbitos por infecções respiratórias não covid-19 em crianças menores de 5 anos no Brasil de 2002 a 2022. Espera-se que sirva como linha de base para estudos futuros, após a adoção em larga escala das novas tecnologias de prevenção de vírus sincicial respiratório.

## Métodos 

### Delineamento 

Trata-se de estudo de série temporal das taxas de mortalidade por infecção respiratória não covid-19 em crianças menores de 5 anos de idade no Brasil, de 2002-2022, além da descrição desses óbitos. 

### Contexto 

O Brasil é um país dividido em cinco regiões (Norte, Nordeste, Centro-Oeste, Sudeste e Sul) com 27 Unidades Federativas. De acordo com o Instituto Brasileiro de Geografia e Estatística, a população era de 214.828.540 habitantes em 2022. Nesse mesmo ano, 2.561.922 crianças nasceram vivas, e a população de menores de 5 anos era de 14.675.523 crianças, o que correspondeu a 1,19% e 6,83% da população geral, respectivamente (10,11).

### Participantes 

Consideraram-se os óbitos de crianças menores de 5 anos por infecção respiratória notificadas no Sistema de Informações sobre Mortalidade com causa básica registrada com os seguintes códigos da 10ª revisão da Classificação Internacional de Doenças: B97 (agentes virais como causa de doenças classificadas em outros capítulos), J00-J06 (infecções agudas das vias aéreas superiores), J09-J18 (influenza e pneumonia), J20-J22 (outras infecções respiratórias agudas das vias aéreas inferiores) ou J40 (bronquite não especificada como aguda ou crônica). Não foram incluídos óbitos com causa básica relacionada à covid-19.

### Variáveis 

Os óbitos por infecções respiratórias não covid-19 foram descritos segundo a data de óbito, a naturalidade, a idade no óbito em anos (0, 1, 2, 3, 4), o sexo (feminino, masculino, ignorado), o peso ao nascer (<2,5 mil g; ≥2,5 mil g), a raça/cor da pele (amarela, branca, indígena, negra [pretos e pardos], ignorado), o local de ocorrência do óbito (hospital, outro serviço de saúde, domicílio, outros), a faixa etária da mãe (<15, 15-19, 20-24, 25-29, 30-34, ≥35 anos), a escolaridade materna (nenhuma, 1-3, 4-7, 8-11, 12+ anos, ignorado), a quantidade de filhos vivos (≤1, 2-3, >3, ignorado), o tipo de parto (cesáreo, vaginal, ignorado), o tipo de gravidez (única, dupla, tripla e mais, ignorada) e a semana de gestação (≤36 semanas, >36 semanas, ignorado).

Os indicadores epidemiológicos calculados foram os descritos a seguir.

- Taxa de mortalidade por infecções respiratórias não covid-19 em menores de 5 anos no Brasil/região: óbitos por infecções respiratórias não covid-19 em menores de 5 anos no Brasil/região dividido pelo número de nascidos vivos no Brasil/região multiplicado por 1 mil.
- Mortalidade proporcional por infecções respiratórias não covid-19 em menores de 5 anos no Brasil/região: óbitos por infecções respiratórias não covid-19 em menores de 5 anos no Brasil/região dividido pelo total de óbitos em menores de 5 anos no Brasil/região multiplicado por 100.

### Fontes de dados

Os dados do Sistema de Informações sobre Mortalidade foram obtidos junto à Plataforma de Ciência de Dados aplicada à Saúde da Fundação Oswaldo Cruz (12). Essa Plataforma utilizou dados anonimizados e disponibilizados pelo Departamento de Informática do Sistema Único de Saúde, realizando o tratamento e enriquecimento por meio de metodologia própria (13). 

Foram utilizados também os números de nascidos vivos disponíveis no Sistema de Informações sobre Nascidos Vivos no site do Departamento de Informática do Sistema Único de Saúde, pelo Tabnet, para Brasil e Unidades da Federação segundo os anos do estudo (11). O banco de dados e os códigos de análise utilizados na pesquisa estão disponíveis em: https://drive.google.com/drive/folders/17aHqCAwu3j2qjjb97wUd3jbeIV4Vq_XF?usp=sharing (14).

### Métodos estatísticos

A estatística descritiva dos óbitos deste estudo foi estimada por meio de frequências absolutas e relativas das variáveis selecionadas. Os indicadores epidemiológicos citados foram calculados anualmente segundo Brasil e regiões geográficas. Para as análises das séries temporais, as taxas de mortalidade por infecções respiratórias não covid-19 em menores de 5 anos foram calculadas mensalmente para o Brasil e foi aplicado o modelo de Trigonometric Box-Cox Transformation ARMA errors Trend and Seasonal Components (TBATS) do pacote Forecast no ambiente de computação R (versão 4.3.1, R Foundation for Statistical Computing, Viena, Áustria. Disponível em: https://www.R-project.org/) (15). 

O modelo TBATS foi considerado adequado após avaliação da autocorrelação dos resíduos por meio de gráfico de função de autocorrelação e teste de Ljung-Box (16). Além disso, o pacote Seastests foi utilizado para identificar tendências e sazonalidade por meio do teste de Friedman (17). Utilizou-se o método de análise de séries temporais interrompidas devido à ocorrência da pandemia de covid-19 em 2020 e 2021 (17). 

Foram analisadas as taxas mensais de óbitos por infecções respiratórias não covid-19 em menores de 5 anos (variável dependente) no período 2002-2022 (variável independente), com padrão de sazonalidade de 12 meses. Os anos da série anteriores à pandemia de covid-19 foram de 2002 a 2019, e o período pandêmico, entre 2020 e 2021. O ano de 2022 foi considerado pós-pandemia de covid-19 (18). 

Para descrever as tendências lineares, foi admitida significância estatística com p-valor<0,05. Estimou-se a variação percentual anual (VPA) do período para apresentar a tendência da série pelo cálculo do logaritmo natural dos valores das taxas de mortalidade mensais de infecções respiratórias não covid-19 em menores de 5 anos de modo a linearizar a tendência. Foi aplicado um modelo de regressão linear, e calculou-se a VPA por meio da seguinte equação (18): VPA=(exp(β)−1)*100, em que β é o coeficiente da regressão. 

Os intervalos de confiança de 95% da VPA também foram estimados por IC_vpa_=(exp(β±t_α_/_2_,_df_×SE(β))−1*100, em que t_α_/_2_,_df_ é o valor crítico do teste T com 5% de significância e df são os graus de liberdade do modelo (número de observações menos número de parâmetros). 

A tendência foi considerada crescente para valores positivos de VPA, decrescente para valores negativos e estável quando os resultados não foram estatisticamente relevantes.

## Resultados 

No período 2002-2022, 52.015 óbitos em crianças menores de 5 anos no Brasil, cuja causa básica foi infecção respiratória não covid-19, foram registrados no Sistema de Informações sobre Mortalidade. As maiores frequências de óbitos foram observadas em: sexo masculino (54,2%; n=28.174), negros (46,0%; n=23.936), menores de 1 ano (66,9%; n=34.792) e hospital como local de ocorrência do óbito (85,0%; n=44.197) (Tabela 1). Houve maior percentual de brancos no Sudeste (54,4%; n=9.640) e no Sul (81,9%; n=3.421). Assim como a variável peso ao nascer, as variáveis maternas faixa etária, escolaridade, tipo de gravidez, semanas de gestação, tipo de parto e quantidade de filhos vivos estavam com mais de 50,0% da informação ignorada.

**Tabela 1:** Características dos óbitos por infecções respiratórias não covid-19 em menores de 5 anos de idade. Brasil e regiões, 2002-2022 (n=52.015)

Variáveis	Brasil n (%)	Norte n (%)	Nordeste n (%)	Centro-Oeste n (%)	Sudeste n (%)	Sul n (%)
Sexo						
Feminino	23.813 (45,8)	4.366 (45,7)	7.609 (45,7)	1.787 (45,6)	8.164 (46,1)	1.887 (45,2)
Masculino	28.174 (54,2)	5.173 (54,2)	9.031 (54,2)	2.131 (54,4)	9.551 (53,9)	2.288 (54,8)
Ignorado	28 (0,1)	7 (0,1)	17 (0,1)	2 (0,0)	2 (0,0)	0 (0,0)
**Idade** (anos)						
0	34.792 (66,9)	6.392 (67,0)	11.204 (67,3)	2.550 (65,0)	11.814 (66,7)	2.832 (67,9)
1	9.156 (17,6)	1.710 (17,9)	2.934 (17,6)	716 (18,3)	3.077 (17,4)	719 (17,2)
2	3.953 (7,6)	736 (7,7)	1.197 (7,2)	344 (8,8)	1.374 (7,7)	302 (7,2)
3	2.418 (4,6)	438 (4,6)	763 (4,6)	190 (4,8)	847 (4,8)	180 (4,3)
4	1.696 (3,3)	270 (2,8)	559 (3,3)	120 (3,1)	605 (3,4)	142 (3,4)
**Raça/cor da pele**						
Amarela	111 (0,2)	22 (0,2)	36 (0,2)	7 (0,2)	41 (0,2)	5 (0,1)
Indígena	2.482 (4,8)	1.393 (14,6)	251 (1,5)	659 (16,8)	74 (0,4)	105 (2,5)
Branca	20.452 (39,3)	1.986 (20,8)	3.972 (23,8)	1.433 (36,5)	9.640 (54,4)	3.421 (81,9)
Negra	23.936 (46,0)	5.737 (60,1)	9.521 (57,2)	1.571 (40,1)	6.638 (37,5)	469 (11,3)
Ignorado	5.034 (9,7)	1.393 (4,3)	2.877 (17,3)	250 (6,4)	74 (7,5)	175 (4,2)
**Local de ocorrência do óbito**						
Hospital	44.197 (85,0)	7.914 (82,9)	14.010 (84,1)	3.274 (83,5)	15.428 (87,1)	3.571 (85,5)
Outro serviço de saúde	1.931 (3,7)	161 (1,7)	351 (2,1)	154 (3,9)	1.149 (6,5)	116 (2,8)
Domicílio	4.079 (7,8)	921 (9,6)	1.598 (9,6)	321 (8,2)	891 (5,0)	348 (8,3)
Outros locais	1.641 (3,2)	487 (5,1)	632 (3,8)	162 (4,1)	231 (1,3)	129 (3,1)
Ignorado	167 (0,3)	63 (0,7)	66 (0,4)	9 (0,2)	18 (0,1)	11 (0,3)

A taxa de mortalidade por infecções respiratórias não covid-19 em menores de 5 anos no Brasil em 2002 foi 1,22 óbito/1 mil nascidos vivos e em 2022 foi 0,87 óbito/1 mil nascidos vivos. Em 2020, foi registrada a menor taxa de mortalidade (0,33/1 mil nascidos vivos) (Tabela 2). O Norte teve a maior taxa de mortalidade por 1 mil nascidos vivos em todo o período, com o maior registro em 2004 (1,86 óbito/1 mil nascidos vivos). O Sul teve a menor taxa com 0,13 óbito/1 mil nascidos vivos em 2020 (Figura 1).

**Tabela 2:** Taxa de mortalidade e mortalidade proporcional por infecções respiratórias não covid-19 em menores de 5 anos de idade. Brasil, 2002-2022 (n=52.015)

Ano	Taxa de mortalidade em menores de 5 anos por 1 mil nascidos vivos	Mortalidade proporcional (%) em menores de 5 anos	Mortalidade proporcional (%) em menores de 5 anos, excluídos óbitos neonatais (n=50.159)
2002	1,22	5,68	12,86
2003	1,27	6,01	13,40
2004	1,21	6,09	14,02
2005	1,11	5,91	13,33
2006	1,12	6,01	14,77
2007	1,00	5,67	13,48
2008	0,92	5,45	13,34
2009	0,95	5,71	13,88
2010	0,78	4,98	12,58
2011	0,84	5,50	13,55
2012	0,80	5,37	13,23
2013	0,85	5,71	13,31
2014	0,74	5,18	13,03
2015	0,63	4,65	12,22
2016	0,72	5,11	12,64
2017	0,61	4,46	11,51
2018	0,67	4,96	12,43
2019	0,73	5,29	13,78
2020	0,33	2,66	6,93
2021	0,43	3,31	8,71
2022	0,87	6,11	14,92

**Figura 1:** Taxa de mortalidade por infecções respiratórias não covid-19 em menores de 5 anos de idade. Brasil e regiões, 2002-2022

No período 2002-2022, a mortalidade por infecções respiratórias não covid-19 em crianças no Brasil apresentou sazonalidade anual, com elevação da taxa de mortalidade em meados de março, atingindo o pico em maio e decréscimo em julho. Também foi observada elevação na taxa em janeiro seguida de queda. O padrão sazonal foi mantido durante o período pré-pandemia de covid-19 e, a partir de 2020, a sazonalidade anual foi observada, porém com picos menores nas taxas de óbitos (Figura 2). 

**Figura 2:** Série temporal da taxa mensal de mortalidade (por 1 mil nascidos vivos) por infecções respiratórias não covid-19 em menores de 5 anos de idade: (A) observações; (B) tendência; (C) sazonalidade; (D) aleatoriedade. Brasil, 2002-2022

Apresentaram-se a distribuição da taxa mensal de mortalidade por infecções respiratórias não covid-19 (Figura 2A), a tendência da taxa de mortalidade (Figura 2B) e o padrão sazonal da ocorrência da taxa de mortalidade (Figura 2C). A aleatoriedade (ou ruído branco) especificou o comportamento da taxa de mortalidade por infecções respiratórias não covid-19 em menores de 5 anos, que não foi explicado pela tendência ou pela sazonalidade (Figura 2D). 

No período 2002-2022, foi observada redução média anual de 0,01 na taxa de mortalidade por infecções respiratórias não covid-19 em menores de 5 anos, sendo essa tendência avaliada como estatisticamente significativa (p-valor 0,034). Considerando a análise de série temporal interrompida (interrupção nos anos pandêmicos de 2020 e 2021), houve dois períodos de estudo: 2002-2019 e o ano de 2022. 

Observou-se tendência de redução da taxa de mortalidade no período 2002-2019 (VPA -0,37%; IC95% -0,43; -0,31). Os anos pandêmicos de 2020 e 2021 apresentaram as menores taxas de mortalidade da série, afetando o comportamento da taxa de mortalidade ao longo dos anos analisados. Em 2022 (pós-pandemia), não houve variação significativa na taxa de mortalidade por infecções respiratórias não covid-19 em menores de 5 anos (VPA -1,41%; IC95% -8,74; 6,52) (Tabela 3). 

**Tabela 3:** Valores de variação percentual anual (VPA) e intervalo de confiança 95% (IC95%) para a taxa de mortalidade por infecções respiratórias não covid-19 em menores de 5 anos de idade. Brasil, 2002-2022 (n=52.015)

Período (anos)	VPA	IC95%
2002-2019	-0,37%	-0,43; -0,31
2022	-1,41%	-8,74; 6,52

A mortalidade proporcional no período do estudo variou de 2,7% em 2020 a 6,1% em 2022 para todo o país (Tabela 2). As regiões com menor e maior mortalidade proporcional por infecções respiratórias não covid-19 no período do estudo foram a Sul em 2020 (1,3%) e a Norte em 2013 (9,6%) (dados não apresentados em tabelas e figuras). Quando excluído o período neonatal, a mortalidade proporcional em crianças de 28 dias a 5 anos variou em torno de 13,0% por ano, sendo o menor valor 11,5% em 2017 e o maior 14,9% em 2022. Nos anos pandêmicos, essa mortalidade foi 6,9% em 2020 e 8,7% em 2021 (Tabela 2).

## Discussão 

No período 2002-2022, no Brasil, houve maior concentração de óbitos por infecções respiratórias em crianças menores de 1 ano e negras. As taxas de mortalidade por infecções respiratórias não covid-19 em menores de 5 anos foram maiores no Norte do país e menores no Sul em todos os anos estudados. A mortalidade proporcional em crianças de 28 dias a menores de 5 anos foi acima de 13%. Em todo o período, foi identificada sazonalidade anual no país, havendo tendência de queda na taxa de mortalidade por infecções respiratórias.

Este estudo mostrou que a mortalidade por infecção respiratória em menores de 5 anos no Brasil encontrou-se dentro da meta de menos de 3 mortes por 1 mil nascidos vivos até 2025 estabelecida pela Organização Mundial da Saúde e pelo Fundo das Nações Unidas para a Infância no Plano de Ação Global para a Prevenção e Controle da Pneumonia e Diarreia, que tem por objetivo acabar com as mortes evitáveis por pneumonia (19). Observou-se queda significativa da mortalidade por infecção respiratória em menores de 5 anos no Brasil de 2002 a 2019, período prévio à pandemia de covid-19. A queda dos óbitos por infecções respiratórias não covid-19 foi ainda mais pronunciada durante a pandemia de covid-19 em 2020 e 2021, provavelmente refletindo o efeito das medidas não farmacológicas de prevenção de covid-19, como fechamento de escolas, isolamento social e uso disseminado de máscaras em ambientes públicos. Em 2022, houve aumento da taxa de mortalidade em menores de 5 anos para níveis próximos ao observado em 2019, uma vez que essas medidas foram suspensas. 

Estimativas de estudos da carga global de doenças mostraram queda significativa de 67% e 77% dos óbitos por infecções respiratórias entre menores de 5 anos, de 1990 a 2019, em todo o mundo e na América Latina, respectivamente (20). Dados de 2021 estimaram taxa de mortalidade por infecção respiratória em menores de 5 anos para 100 mil habitantes de 76 para o mundo e 25 para a América Latina (20). No Brasil essa redução foi de 96% de 1980 a 2021, passando de 302 para 11 óbitos em menores de 5 anos por pneumonia e outras infecções respiratórias por 100 mil habitantes, respectivamente (20). Grande parte dessa redução no mundo e no Brasil foi atribuída a medidas como estímulo à amamentação exclusiva até os 6 meses de idade, aumento da cobertura vacinal contra *Haemophilus influenzae* e *Streptococcus pneumoniae*, aumento do acesso aos serviços de saúde e manejo clínico adequado dos casos de infecção respiratória (21).

Apesar de todo esse avanço histórico, a mortalidade por infecção respiratória está relacionada com níveis de pobreza e desigualdade (20). A mortalidade por doença respiratória constitui a segunda causa de óbito em menores de 5 anos no mundo e era a primeira causa de óbito na infância excluído as causas neonatais no Brasil em 2019 (1,22).

Com relação à mortalidade proporcional por infecção respiratória, esta compõe mais de 10% dos óbitos excluído o período neonatal. No Brasil, não houve redução da mortalidade proporcional durante o período estudado, exceto no período pandêmico, o que mostra redução proporcionalmente menor dos óbitos por infecção respiratória quando comparado a outras causas. 

A maioria dos óbitos ocorreu em crianças negras. A mortalidade infantil decorrente de causas evitáveis afeta mais crianças negras e esse fator aparece também associado à baixa escolaridade materna (23). No Brasil, entre crianças nascidas de 1º de janeiro de 2012 a 31 de dezembro de 2018, crianças negras apresentaram risco 39% maior de morrer antes dos 5 anos em comparação às brancas, além de aumento de 78% no risco de óbito por pneumonia (24). Fatores como acesso limitado a serviços de saúde, condições precárias de saneamento básico e maior incidência de desnutrição foram apontados como contribuintes para essa disparidade. 

As estimativas mais fortes de associação entre raça materna/cor da pele e mortalidade infantil foram encontradas para influenza e pneumonia. As maiores disparidades étnico-raciais foram observadas em crianças de 1 a 4 anos para diarreia, desnutrição, gripe e pneumonia (24). A sobrevivência de pós-neonatos e crianças de 1 a 4 anos foi mais adversamente afetada pelas condições de vida da família, renda, educação, saneamento básico, acesso à água limpa e acesso a serviços de saúde (24-26).

O Norte destacou-se por alcançar os maiores valores ao longo de toda a série histórica, ao contrário do Sul. No Sul, houve o maior percentual da população que referiu ter realizado pelo menos uma consulta médica e odontológica durante o ano quando comparado às outras regiões do país (27). O Norte enfrentou desafios como menor cobertura de atenção básica, maior distância dos serviços de saúde especializados e escassez de leitos pediátricos e de unidade de terapia intensiva (28). Esses fatores limitaram o manejo adequado de casos graves de infecções respiratórias. Por outro lado, o Sul se beneficiou de indicadores de desenvolvimento humano mais elevados, maior número de hospitais bem equipados e melhor distribuição de profissionais de saúde, o que contribuiu para a redução das taxas de mortalidade (28,29).

Observou-se sazonalidade dos óbitos por doenças respiratórias em todos os anos da série histórica, que se iniciou em março, com pico em maio e decréscimo em julho. Esse padrão sazonal de óbitos por doenças respiratórias coincidiu com a sazonalidade do vírus sincicial respiratório, que é o principal agente de infecção respiratória em menores de 5 anos, sobretudo em menores de 1 ano (30).

A interpretação dos resultados deste estudo deve levar em consideração as limitações relacionadas ao uso de dados secundários que podem apresentar sub-registros e erros de digitação. Houve percentual elevado de variáveis com categorias ignoradas, como peso ao nascer da criança e relacionadas à mãe (faixa etária, escolaridade, tempo de gestação, tipo de parto e quantidade de filhos vivos). Essas são variáveis importantes, porém não foi possível interpretá-las, podendo serem subestimadas ou superestimadas. Dessa forma, torna-se importante a sensibilização dos profissionais responsáveis, para que todas as variáveis da declaração de óbito sejam preenchidas. 

É possível que alguns óbitos por doenças respiratórias não tenham sido corretamente classificados na declaração de óbito e no Sistema de Informações sobre Mortalidade; isso pode levar à subestimação do número de óbitos. Porém, considerando que a série de dados é longa e que tem ocorrido melhorias no Sistema de Informações sobre Mortalidade nos últimos anos, pode ser que, nos anos mais recentes, os óbitos estejam mais bem classificados do que nos anos anteriores.

Em conclusão, neste estudo foram identificadas as taxas de mortalidade por infecção respiratória no Brasil utilizando dados secundários do sistema nacional de vigilância do óbito. A ocorrência dos óbitos parece seguir uma sazonalidade que coincide com a do vírus sincicial respiratório, principal agente de infecção respiratória grave em menores de 5 anos. Apesar da redução das taxas de mortalidade por essas doenças ao longo da série histórica, não houve redução da mortalidade proporcional, o que ressalta que novas estratégias são necessárias para prevenir e reduzir os óbitos por infecções respiratórias no sistema de saúde brasileiro. 

Estes resultados ganham relevância, pois servem de base para a avaliação do impacto de novas tecnologias de prevenção das infecções por vírus sincicial respiratório incorporadas ao Sistema Único de Saúde: uma vacina para gestantes e um novo anticorpo monoclonal contra o vírus sincicial respiratório. Observou-se maior número de óbitos em crianças negras e no Norte do país, que pode refletir desigualdades na prevenção e no tratamento dessas infecções. Estes dados são apenas descritivos, por isso estudos analíticos futuros são necessários para avaliar o risco dessas variáveis.
